# UVMS task-priority planning framework for underwater task goal classification optimization

**DOI:** 10.3389/fnbot.2022.982505

**Published:** 2022-11-28

**Authors:** Yu-er Gao, Xiaohui Zhang, Yan Su, Jinbao Wang, Qihang Yang, Wenqi Bai, Songnan Yang

**Affiliations:** ^1^Department of Information and Control Engineering, Xi'an University of Technology, Xi'an, China; ^2^Hanjiang-Weihe River Valley Water Diversion Project Construction Co., LTD., Xi'an, China

**Keywords:** underwater vehicle manipulator system, task prioritization strategy, motion planning, trajectory optimization, nonlinear optimization

## Abstract

This paper presents a task prioritization strategy based on a generic underwater task goal classification transformation for multitasking underwater operational tasks: attitude control, floating manipulation, collision-free motion, especially optimizing trajectory of the end-effector of an underwater vehicle manipulator system (UVMS) in a complex marine environment. The design framework aims to divide the complex underwater operational tasks into UVMS executable generic task combinations and optimize the resource consumption during the whole task. In order to achieve the corresponding underwater task settings, the system needs to satisfy different task scheduling structures. We consider the actual application scenarios of the operational goals and prioritize and define each category of task hierarchy accordingly. Multiple tasks simultaneously enable fast adaptation to UVMS movements and planning to complete UVMS autonomous movements. Finally, an underwater vehicle manipulator system implements the task prioritization planning framework for a practical scenario with different constraints on different goals. We quickly and precisely realize the interconversion of different tasks under goal constraints. The autonomous motion planning and real-time performance of UVMS are improved to cope with the increasing operational task requirements and the complex and changing practical engineering application environments.

## Introduction

As the largest ecosystem on Earth, the ocean regulates not only global climate change but also supports global economic development by providing humans with productive resources such as protein, water, and energy. In the past decades, understanding and developing the oceans require various high technologies and equipment, including underwater robots, which have been the focus of attention worldwide. In particular, the underwater vehicle manipulator system (UVMS) plays a pivotal role in national projects [RAUVI (Sanz et al., 2011) or ARCHROV (Casalino et al., 2012)] and European projects [FP7 STREP TRIDENT (Sanz et al., 2010), PANDORA (Heshmati-Alamdari et al., 2018), MORPH, Eurofelts2 (Olguin-Diaz et al., 2013), etc.] about underwater robots, which indicates that the emergence as one of the most powerful tools for human research and exploitation activities of marine resources.

Due to the redundancy of degrees of freedom, nonlinearity, strong coupling, time variation, high dimensionality, low bandwidth of sensor data acquisition, and interference by hydrodynamic forces, the system design, autonomous control, and operation planning of UVMS has become a challenging topic in the field of underwater robotics at home and abroad. Since the underwater environment is different from the surface, the realization of the control task of the robot system becomes more difficult. The control problem of UVMS as a redundant system is challenging to be solved completely. However, the redundancy characteristics of the system can be explored reasonably to perform multiple tasks simultaneously and ensure that each task is eventually completed. Therefore, it is necessary to coordinate the motions of the body and the manipulator when meeting the operational task requirements (Fujie et al., 2020). The most commonly used methods for motion planning are the minimum parametric solution method, the weighted pseudo-inverse method, the gradient projection method, and the task prioritization method to achieve UVMS motion planning (Whitney, 1969). On top of this method, some scholars have improved and extended it by introducing the weight matrix into the robot body and manipulator's joints to achieve weighted parametric optimality in robot configuration. Gianluca Antonelli in Italy proposed a task priority-based planning method for UVMS motion, which sets the primary and secondary tasks, prioritizes to ensure the completion of the primary task, and completes the secondary tasks as much as possible under the premise of completing the primary task (Han et al., 2011). Task prioritization methods are used to decide the order of task execution according to the task priority level when multiple tasks are in conflict and are often used to solve redundancy problems. For example, Antonelli and Chiaverini (1998), Cieslak et al. (2015), Changmi (2022), and Gancet et al. (2016) used this approach. Tang et al. (2017) proposed an acceleration level task priority redundancy decomposition method. Simetti et al. (2018) proposed a task priority approach that can be applied to different scenarios in UVMS. The multitask weight gradient method has also been used for secondary task weight assignment (Wang et al., 2017). Sotiropoulos et al. (2015) proposed a fast motion planning algorithm for UVMS in semi-structured environments. Youakim et al. (2017) used different motion planning methods to simulate and analyze the motion of an underwater manipulator, solving the problem of “which planner to choose”. Depending on the specific situation, different strategies are needed for the dual-arm problem (Moe et al., 2014; Bae et al., 2018) and the cooperative operation problem (Xuefeng and Xinqian, 2000; Chang, 2004; Conti et al., 2015; Simetti and Casalino, 2016). Some researchers have developed analysis software packages, such as UWSim (Prats et al., 2012) and MoveIt! For example, it is guaranteed that the end position pose of UVMS reaches the desired value, and then constraints such as system energy consumption or manipulator limit are implemented. Subsequently, based on this algorithm, researchers introduced the fuzzy theory to adjust the parameters online for the problem of optimal allocation of primary and multiple secondary tasks (Antonelli and Chiaverini, 2000) to improve task self-adaptability and achieve multitask planning. The literature (Podder and Sarkar, 2000) decomposed the overall motion into the body and the manipulator motion based on the response differences between the body and the manipulator systems in the dynamics model. Based on the UVMS kinetic model, Huang Hai et al. of Harbin Engineering University proposed pairwise optimization combined with a genetic algorithm for trajectory planning of UVMS motion (Huang et al., 2016), which obtained a set of hull positions and manipulator joint angles by genetic, crossover, and variation operations. If the set of sequences satisfied the error range, then the adaptive function was compared and continuously iterated to obtain the optimal global solution.

However, most of the above studies consider a single task during planning. Few have come to deal with task coordination and planning of transitions between tasks in underwater vehicle manipulator system. Tasks can be kinematic (position) or kinetic (force) goals for robot motion control. The ability of a robot to accomplish a goal depends on its physical limitations and surrounding environmental obstacles. Nakamura et al. (1987) introduced the concept of task prioritization associated with the inverse problem of redundant degree manipulator kinematics to determine joint motions with sequential tasks. A general framework for managing multiple tasks of highly redundant robotic systems was proposed in Siciliano and Slotine (1991), but only equality tasks were considered. Researchers tended to transform inequality tasks into equality tasks with the highest priority (Sentis and Khatib, 2005; Mansard and Chaumette, 2007), which could lead to discontinuities. Mansard et al. (2009) used a weighted solution to overcome this drawback with a limited number of inequalities. Inspired by the sequential least-squares formulation of the classical task framework (Jin, 1996), Kanoun et al. (2011) extended the task prioritization framework to inequality tasks. They applied the algorithm to the humanoid robot HRP-2. While much work has been done at the UVMS control level, many scholars have proposed many approaches to the trajectory tracking control of UVMS. Han et al. considered the effect of external disturbances and proposed nonlinear H-optimal control with disturbance observer for implementing tracking control of UVMS in Ref. (Han and Chung, 2008). Mohan et al. proposed an indirect adaptive control method based on the Kalman filter for autonomous operation of UVMS, which overcomes the drawbacks of existing disturbance observer and direct adaptive control. The method can target the consideration of load compensation, underwater currents, or external disturbance compensation. Xu et al. proposed a neuro-fuzzy-based intelligent control algorithm for operational control of UVMS. The proposed decentralized neural network compensator was used to estimate UVMS dynamics, which can better cope with load variations and hydrodynamic disturbances (Xu et al., 2012). Olguin-Diaz et al. (2013) proposed a force-motion-based control framework for operational control of UVMS, which does not require prior modeling of UVMS dynamics and applies a second-order model-free sliding mode control method to calculate the control rate in the force-motion framework. Lynch and Ellery (2014) combined feedback and a feedforward approach to achieve robot attitude and position stabilization for effective manipulator control. However, the problem of task planning as the highest level of the control system, task goal assignment, and transformation during under-water tasks performed by UVMS remains a critical issue in the field of robotics. Although studies consider the uncertainty of its operation in the real world, this is an open research area that needs to produce robust planning algorithms.

Optimization is a high-level task in trajectory planning to seek safety when per-forming tasks in a cluttered, dense, and complex underwater environment. Since resources are limited in the marine environment, mission planning needs to be done under the given constraints, solving its underwater constraints, optimization objectives, physical limitations, and resource invocation problems. Therefore, this paper focuses on the UVMS-based task prioritization strategy. Here, we define the hierarchy of underwater control tasks and their priority relationships. The lowest priority optimization objective is a linear constrained quadratic programming problem. Multiple objective functions with priority equation constraints on the tasks define this optimization problem in this study. The main contributions of this paper are twofold: (1) We develop a UVMS-based task prioritization framework to select the priority of the control tasks in terms of the hierarchy of tasks used and the difference in the priority of the objectives. We give the final planning results in task sequences and resource allocation schemes for each phase, which has been achieved successfully. In addition, we deal with the difficulties of multiple task interconversion activation and multi-objective classification definition at one time. Furthermore, the proposed task prioritization framework can extend widely to underwater operations in real scenarios. (2) Based on the highest objective optimization as the lowest priority objective setting, we propose a soft constraint optimization method to avoid possible collisions and expect the optimization trajectory to reach the ideal trajectory to ensure the underwater task execution of high quality.

The rest of this paper is organized as follows. In Section UVMS system modeling, we complete the modeling of the UVMS. Section Task Planning presents the task prioritization framework composition, defines the division of control objectives and control tasks, and provides detailed definitions of the five categories of tasks. Section Trajectory planning explains the trajectory optimization algorithm and proposes an improved collision avoidance method based on soft constraints. In Section Simulation results, we develop code for the task manager and part of the kinematic control layer, do the most important related simulation experiments, and verify the method's effectiveness with two case studies. Finally, Section Conclusions gives conclusions and future work.

## UVMS system modeling

**Figure 1:**
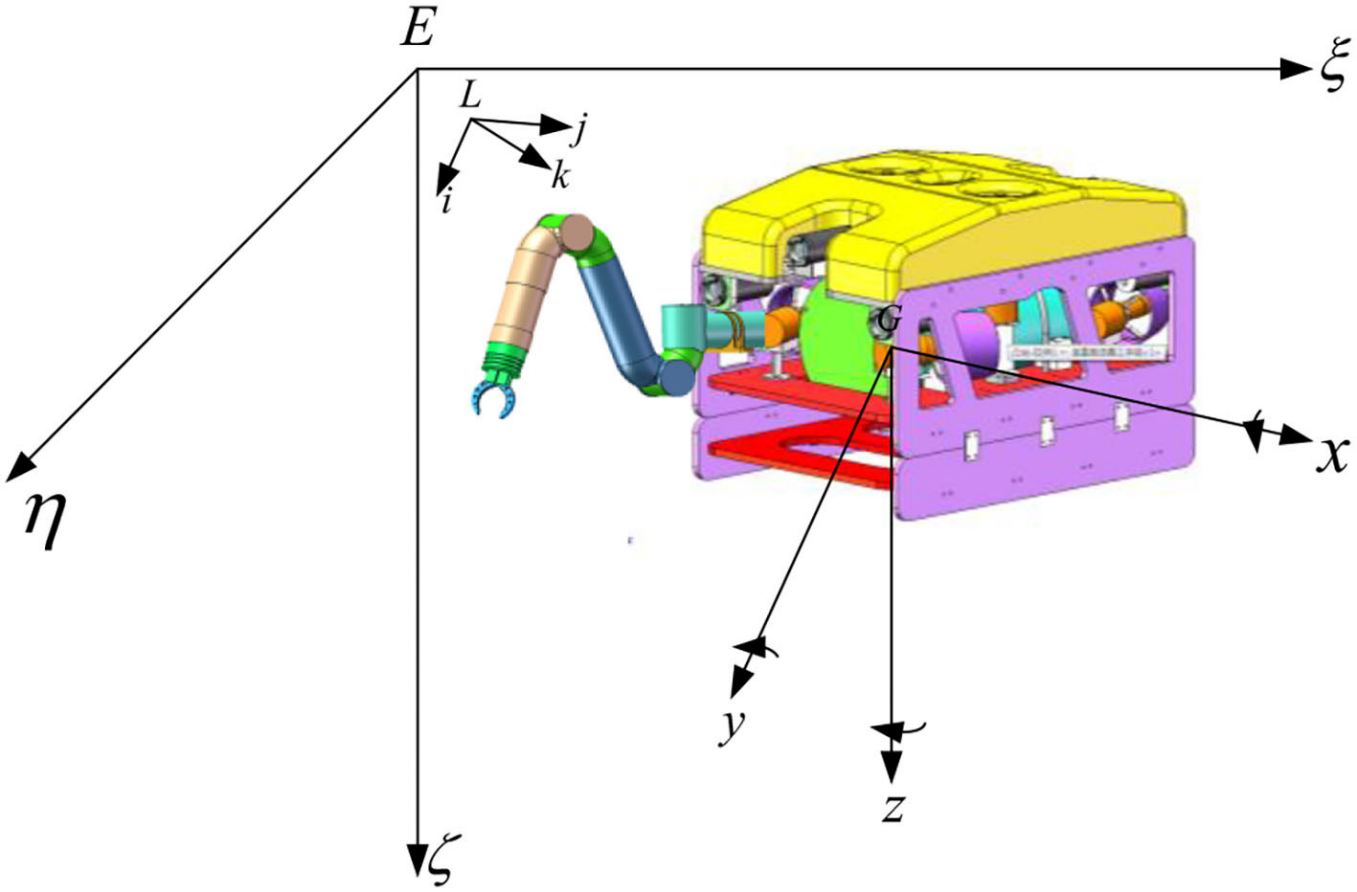
UVMS system structure diagram.

As shown in Figure 1, the UVMS consists of a vehicle and a seven-function manipulator, which is very flexible and well suited for task scenarios with continuous underwater operations.

**Figure 2:**
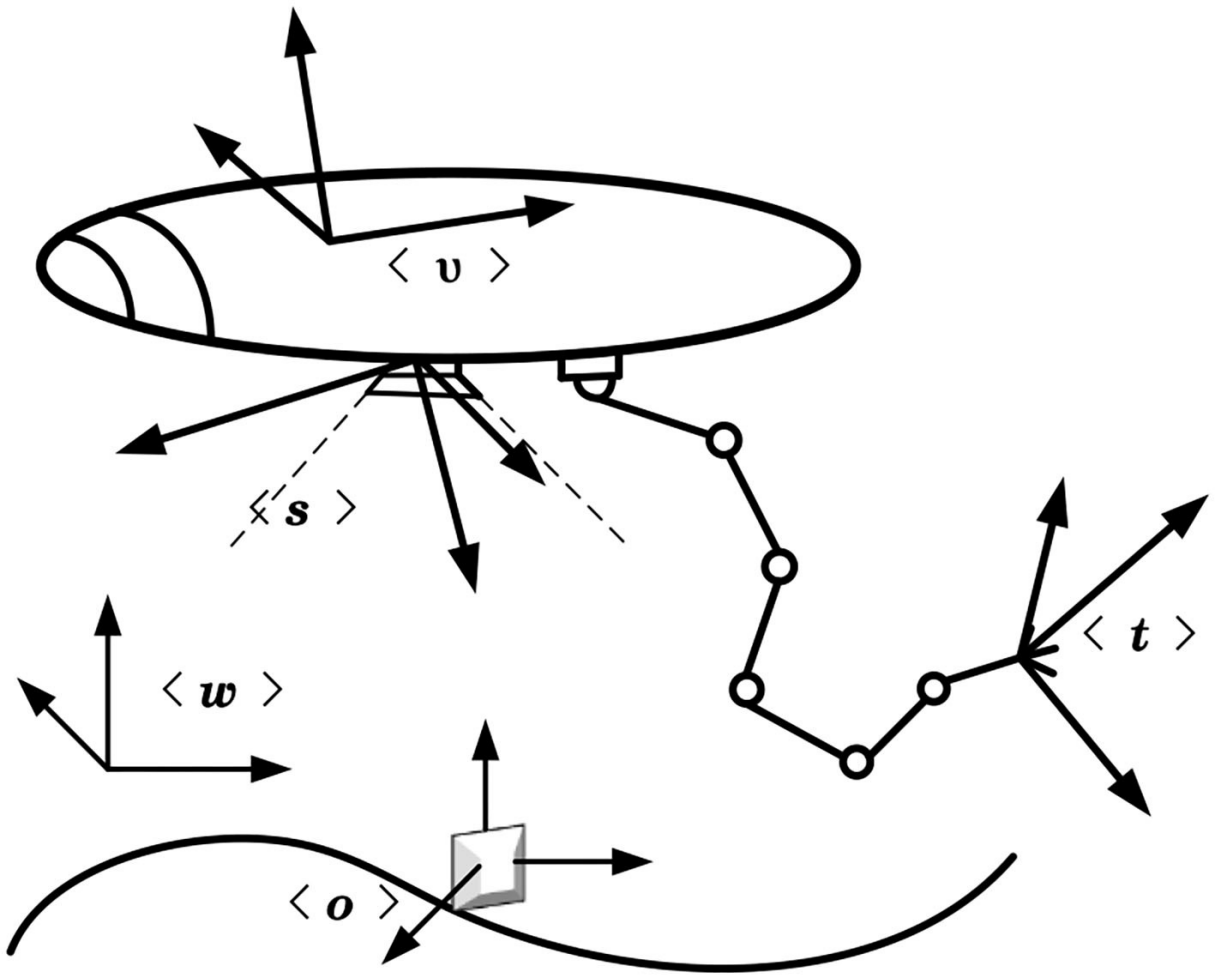
The UVMS and its relevant frames.

As shown in Figure 2, where the fixed inertial frame of the world < w >, the vehicle frame of the UVMS < v >, the sensor frame < s > and the operational target frame < o >, the tool frame < t >.

### Kinematics of UVMS

The kinematics of the end-effector needs to be represented by the whole system, and the system's structure *c* is described by a vector of the parameters of the degrees of freedom of each part of the structure.

(1) $$\begin{array}{l} c=\left[\begin{array}{c} q\\ \eta \end{array}\right] \end{array}$$

where *q* is the description vector of the underwater manipulator

(2) $$\begin{array}{l} q=\left[\begin{array}{c} q_{1}\\ :\\ q_{n} \end{array}\right] \end{array}$$

where η is the description vector of the vehicle

(3) $$\begin{array}{l} \eta =\left[\begin{array}{c} \eta_{1}\\ \eta_{2} \end{array}\right]\in R^{6} \end{array}$$

The relationship between the Euler angular derivative (RPY) and the angular velocity υ_2_ of the vehicle chassis is shown below.

(4) $$\begin{array}{l} \eta_{1}=\left[\begin{array}{c} x\\ y\\ z \end{array}\right],\;\eta_{2}=\left[\begin{array}{c} \phi \\ \theta \\ \psi \end{array}\right] \end{array}$$

(5) $$\begin{array}{l} \overset{.}{y}=\left[\begin{array}{c} \overset{.}{q}\\ \nu \end{array}\right]\;\nu =\left[\begin{array}{c} \nu_{1}\\ \nu_{2} \end{array}\right]\;\begin{array}{c} \nu_{1}{=}^{v}v\\ \nu_{2}{=}^{v}w \end{array} \end{array}$$

where the relationship between the Euler angular rate $\left(\dot{\phi },\;\dot{\theta },\;\dot{\psi }\right)$ and the angular velocity $\left(\begin{array}{c} p,\;q,\;r \end{array}\right)$ of the object in the ontological coordinate system is given by the following equation.

(6) $$\begin{array}{l} \{\begin{array}{l} \dot{\phi }=p+q\left(\sin\phi \tan\theta \right)+r\left(\cos\phi \tan\theta \right)\\ \dot{\theta }=0+q\left(\cos\phi \right)+r\left(-\sin\phi \right)\\ \dot{\psi }=0+q\left(\sin\phi /\cos\theta \right)+r\left(\cos\phi /\cos\theta \right) \end{array} \end{array}$$

Putting it into matrix form yields

(7) $$\begin{array}{l} \left[\begin{array}{c} \dot{\phi }\\ \dot{\theta }\\ \dot{\psi } \end{array}\right]=J\left[\begin{array}{c} p\\ q\\ r \end{array}\right] \end{array}$$

For the angular velocity, the rotation matrix *J*(η) is

(8) $$\begin{array}{l} J\left(\eta \right)=\left(\begin{array}{ccc} 1 & \sin\phi \tan\theta & \cos\phi \tan\theta \\ 0 & \cos\phi & -\sin\phi \\ 0 & \sin\phi /\cos\theta & \cos\phi /\cos\theta \end{array}\right) \end{array}$$

i.e.,

(9) $$\begin{array}{l} \left[\begin{array}{c} \dot{\phi }\\ \dot{\theta }\\ \dot{\psi } \end{array}\right]=\left(\begin{array}{ccc} 1 & \sin\phi \tan\theta & \cos\phi \tan\theta \\ 0 & \cos\phi & -\sin\phi \\ 0 & \sin\phi /\cos\theta & \cos\phi /\cos\theta \end{array}\right)\upsilon_{2} \end{array}$$

Therefore, the following relationship exists between the world frame < w > of the Earth and the vehicle frame < v > of UVMS.

(10) $$\begin{array}{l} {\;}^{w}\dot{x}=J^{v}\dot{y} \end{array}$$

### Dynamics of UVMS

This part is modeled by the Lagrangian method. The kinetic energy of UVMS consists of two parts: the translational kinetic energy and the rotational kinetic energy. The mathematical expression is

(11) $$\begin{array}{l} T_{i}=\frac{1}{2}m_{i}{\dot{r}}_{i}^{2}+\frac{1}{2}J_{i}\omega_{i}^{2}\;\left(i=0,1,2\right) \end{array}$$

where

(12) $$\begin{array}{r} \omega_{0}=\dot{\alpha }Z_{0};\\ \omega_{1}=\left(\dot{\alpha }+{\dot{\theta }}_{1}\right)Z_{1};\\ \omega_{2}=\left(\dot{\alpha }+{\dot{\theta }}_{1}+{\dot{\theta }}_{2}\right)Z_{2} \end{array}$$

The kinetic energy of the entire single-arm system is

(13) $$\begin{array}{l} T=\overset{2}{\underset{i=0}{\sum }}T_{i}\\ \;=\frac{1}{2}m_{0}\left({\dot{x}}_{0}^{2}+{\dot{y}}_{0}^{2}\right)+\frac{1}{2}J_{0}\omega_{0}^{2}+\frac{1}{2}m_{1}\left({\dot{x}}_{1}^{2}+{\dot{y}}_{1}^{2}\right)\\ \;+\frac{1}{2}J_{1}\omega_{1}^{2}+\frac{1}{2}m_{2}\left({\dot{x}}_{2}^{2}+{\dot{y}}_{2}^{2}\right)+\frac{1}{2}J_{2}\omega_{2}^{2}\\ \;=f_{1}\left({\dot{x}}_{0}^{2}+{\dot{y}}_{0}^{2}\right)+f_{2}{\dot{\alpha }}^{2}+f_{3}{\left(\dot{\alpha }+{\dot{\theta }}_{1}\right)}^{2}+f_{4}{\left(\dot{\alpha }+{\dot{\theta }}_{1}+{\dot{\theta }}_{2}\right)}^{2}\\ \;+f_{5}\dot{\alpha }\left({\dot{x}}_{0}\cos\alpha -{\dot{y}}_{0}\sin\alpha \right)+f_{6}\left(\dot{\alpha }+{\dot{\theta }}_{1}\right)\\ \;\left[{\dot{x}}_{0}\cos\left(\alpha +\theta_{1}\right)-{\dot{y}}_{0}\sin\left(\alpha +\theta_{1}\right)\right]\\ \;+f_{7}\left(\dot{\alpha }+{\dot{\theta }}_{1}+{\dot{\theta }}_{2}\right)[{\dot{x}}_{0}\cos\left(\alpha +\theta_{1}+\theta_{2}\right)\\ \;-{\dot{y}}_{0}\sin\left(\alpha +\theta_{1}+\theta_{2}\right)]\\ \;+f_{8}\dot{\alpha }\left(\dot{\alpha }+{\dot{\theta }}_{1}\right)\cos\theta_{1}+f_{9}\dot{\alpha }\left(\dot{\alpha }+{\dot{\theta }}_{1}+{\dot{\theta }}_{2}\right)\cos\left(\theta_{1}+\theta_{2}\right)\\ \;+f_{10}\left(\dot{\alpha }+{\dot{\theta }}_{1}\right)\left(\dot{\alpha }+{\dot{\theta }}_{1}+{\dot{\theta }}_{2}\right)\cos\theta_{2} \end{array}$$

Here, assuming the gravitational potential energy of the system *V* = 0, the Lagrangian function of the system: *L* = *T* − *V*, and using the Lagrangian equation:

(14) $$\begin{array}{l} Q=\frac{d}{d_{t}}\left(\frac{\partial L}{\partial {\dot{q}}_{0}}\right)-\frac{\partial L}{\partial q_{0}} \end{array}$$

where ${\dot{q}}_{0}={\left[\begin{array}{ccccc} {\dot{x}}_{0} & {\dot{y}}_{0} & \dot{\alpha } & {\dot{\theta }}_{1} & {\dot{\theta }}_{2} \end{array}\right]}^{T}$ is the state vector of the system; $Q={\left[\begin{array}{ccccc} 0 & 0 & \tau_{0} & \tau_{1} & \tau_{2} \end{array}\right]}^{T}$ is the control torque matrix of the system.

Substituting into Equation (14) yields the following kinetic equation.

(15) $$\begin{array}{l} D\left(q_{0}\right){\ddot{q}}_{0}+H\left(q_{0},{\dot{q}}_{0}\right){\dot{q}}_{0}=Q \end{array}$$

where *D*(*q*_0_) is the 5^*^5-dimensional symmetric, positive definite mass matrix. $H\left(q_{0},{\dot{q}}_{0}\right)$ is the 5^*^1 dimensional matrix with Koch forces and centripetal forces.

At this point, the dynamics model of the UVMS is established using the Lagrangian modeling method. This dynamical model provides the basis for the task priority control of the underwater manipulator and the underwater robot. Due to the dynamical coupling effect between the manipulator and the base, the motion of the base, and the end effector in free-floating mode is highly dependent on the joint trajectory. Therefore, rational design of task planning solves the problem of multitasking underwater operation tasks such as precise positioning, floating manipulation, or collision-free motion.

## Task planning

### A two-tier framework for task planning

**Figure 3:**
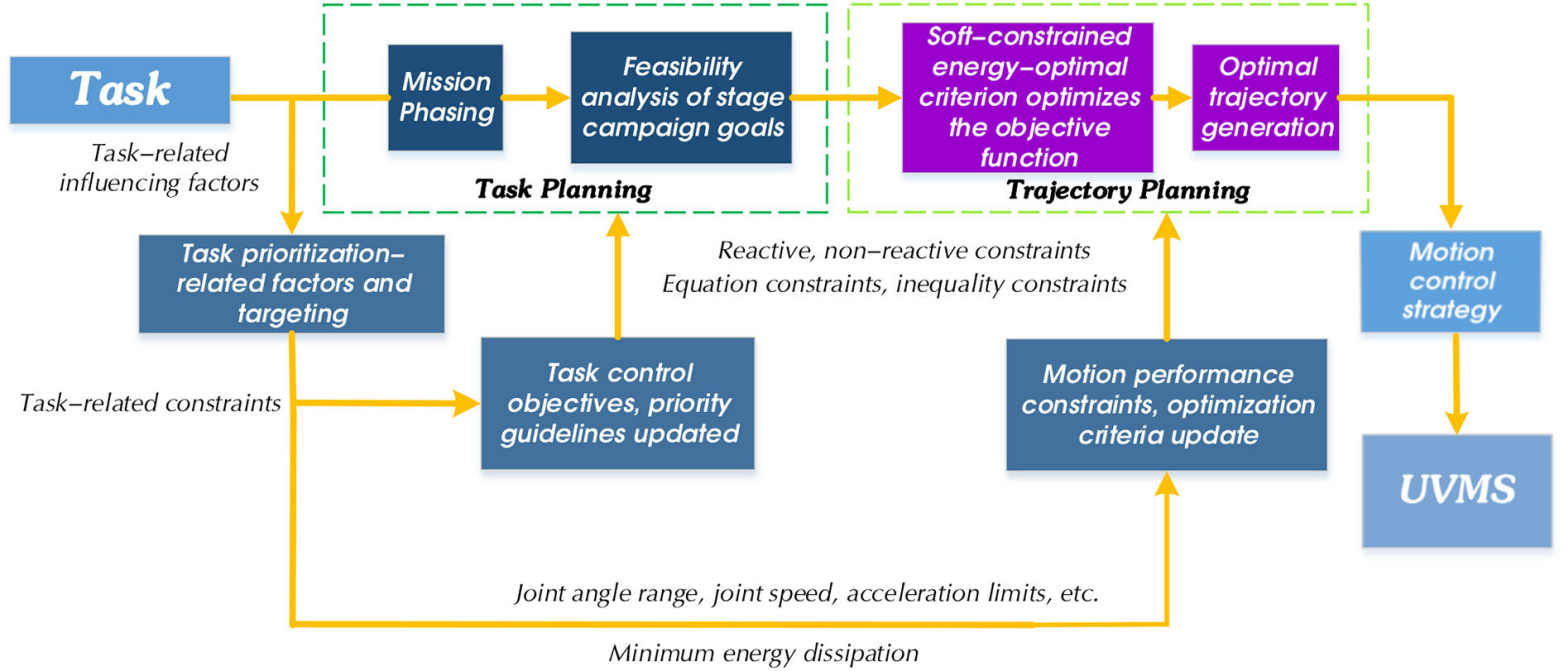
Two-tier framework diagram for task planning.

The UVMS control system in this research consists of task planning, trajectory planning, and motion control. As the top layer of the manipulator control system, task planning is responsible for receiving, analyzing, and disassembling task targets. The purpose is to divide complex task targets into action sequences that the manipulator can directly plan and execute. Due to the diversity of ways for the manipulator to complete tasks, task planning also involves scheduling various types of resources for the manipulator system to optimize resource consumption during the entire task. We give the final planning result through task combination sequences and activation methods. Figure 3 shows the two-layer framework of task planning designed in this paper.

**Figure 4:**
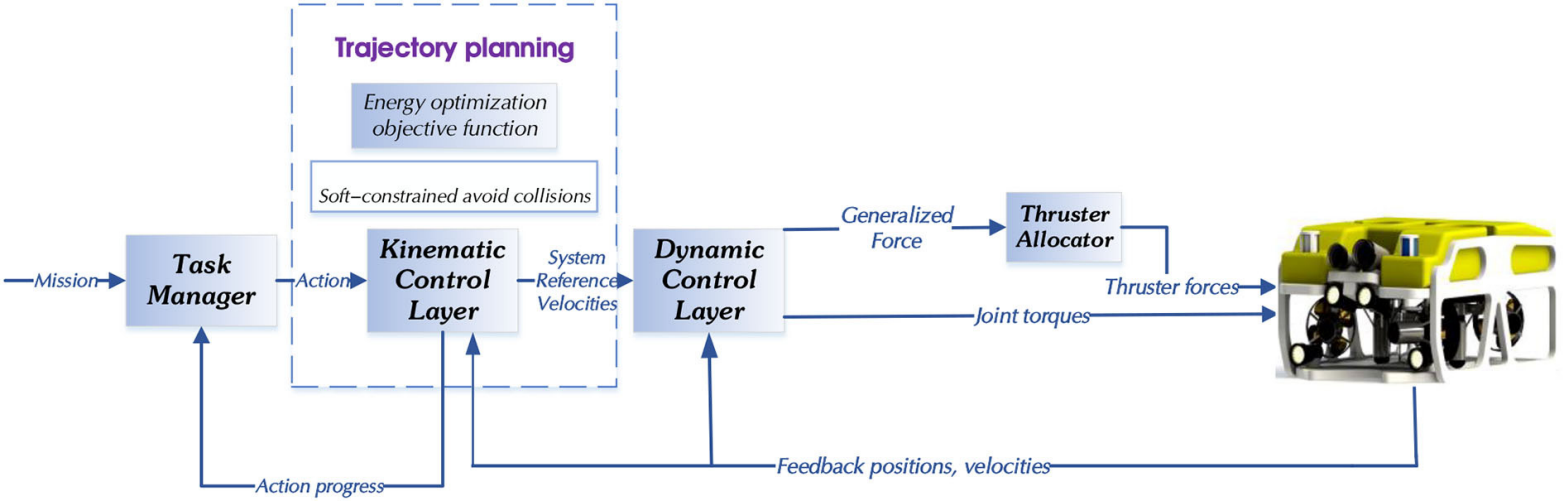
General architecture of the task prioritization approach.

The implementation architecture of the task planning approach proposed in this research is shown in Figure 4.

1. Task Manager: Notifies the Kinematic Control Layer about the actions that must be executed based on the current mission.
2. Kinematic Control Layer: Implements the task priority control framework and generates the system reference velocities. The kinematics control layer mainly manages the end position, joint angle, and speed of UVMS in real-time. For the end-effector, it moves according to the motion trajectory generated by the optimization algorithm designed in this paper and according to the specified motion parameters.
3. Dynamic Control Layer: Tracks the system reference velocities by generating appropriate force/torque references for the vehicle and manipulator.

In the simulation, we develop the code for the Mission Manager and parts of the Kinematic Control Layer.

### Task-priority handling strategy

Considering the task requirements for autonomous UVMS underwater operations, we designed two categories of control tasks.

1. Reactive control task (R): Capable of tracking feedback-generated reference rate $\dot{x}$.
2. Non-Reactive control task (NR): Defined directly in specific task velocity space. Thus, the reference velocity tracked is not generated by feedback.

Also, depending on the type of task, we have designed two different types of objectives.

1. Equality control objective (E): Given by *x*(*c*) = *x*_0_, which is given as a constant equation.
2. Inequality control objective (I): Given by *x*(*c*) ≥ *x*_*min*_ or *x*(*c*) ≤ *x*_max_, which is given as an interval range.

Among them, the objectives can be classified into the following five major categories according to their categories, and this feature assigns priority to each task. The constraint tasks have the highest priority, and the optimization tasks have the lowest priority:

1. Constraints (C): Objective related to the physical constraints of the system.
2. Safety (S): Objectives related to the safety of the robot.
3. Operational Prerequisite (P): Objective that is a prerequisite for the given action.
4. Action Defining (AD): Action-oriented objectives.
5. Optimization (O): Trajectory optimization objective.

Finally, the UVMS task priorities designed in Table 1 are described in conjunction with the definitions of goal types, goal categories, and control tasks elaborated above.

**Table 1:** Control tasks and prioritization.

**Priority**	**Category**	**Description**	**Objective**
1	Safety	[R,I,S]	Minimum Altitude Control (MAC)
2	Prerequisite	[R,I,P]	Horizontal Attitude (HA)
3	Action-defining	[R,E,AD]	Landing Altitude (LA)
4	Prerequisite	[R,E,C]	Alignment Target (AT)
5	Action-defining	[R,E,AD]	Position Control (PC)

[R/NR, I/E, C/S/P/AD] Name of the task/objective.

In Table 1, for task-related factors, the typical underwater tasks of UVMS are first decomposed into the following five basic task categories according to their requirements for autonomous underwater operations.

1. MAC—Assure safe minimum altitude control task [R, I, P]: used to keep the UVMS altitude above a certain threshold.
2. HA—Horizontal attitude task controls the horizontal attitude [R, I, S]: it is critical to maintaining the vehicle-level relative to the whole world frame < w >.
3. LA—Altitude control task, also known as the landing task [R, E, AD]: This action-defining task has the same priority as the vehicle position. The minimum task altitude is not enabled there because we need to land; therefore, UVMS needs to be below a fixed minimum altitude threshold.
4. AT—Target Control Alignment Target Task to Task Alignment [R, I, P]: this is a prerequisite task with a higher priority than the action-defining task. The error range of the inequality is equal to 0.07 m.
5. PC—The vehicle position control task [R, E, AD]: This action-defining task has a lower priority.

Next, we will explain these five types of tasks in detail regarding their task priority selection relationships and reference relationships.

#### Highest priority task “MAC”

The minimum altitude control task “MAC” set in this paper is the security control target. Therefore, its priority must be higher than the actions that define the objective, such as task “PC”. Task “MAC” is the highest priority because avoiding collisions with the seafloor is more important than maintaining the vehicle's horizontal altitude objective during UVMS underwater tasks. This task improves the ability of the UVMS to avoid collisions with the seafloor.

The following Jacobian relationship characterizes the task “MAC”:

(16) $$\begin{array}{l} {\;}^{w}{\dot{x}}_{mac}={J_{mac}}^{v}\dot{y} \end{array}$$

where ${\;}^{w}{\dot{x}}_{mac}\in R^{6}$ represents the task description, $J_{mac}\in R^{6\times 13}$ represents the Jacobian matrix of this task; *J*_*mac*_ has three rows corresponding to the dimensions of the reference velocity ($\dot{x}$_*mac*_), where $\dot{x}$_*mac*_ has only linear components.

(17) $${\;}^{w}{\dot{x}}_{mac}{=}^{w}v_{3\times 1}=\;{\left[\begin{array}{ccc} 0_{3\times 7} & {\;}^{w}R_{v}{\;}_{3\times 3} & 0_{3\times 3} \end{array}\right]}^{v}\;\left[\begin{array}{c} {\dot{q}}_{7\times 1}\\ v_{3\times 1}\\ {\;}^{v}\omega_{3\times 1} \end{array}\right]$$

The task “MAC” is based on an inequality objective, the main goal of which is to ensure that the vehicle maintains its altitude above a certain minimum threshold.

(18) $$\begin{array}{l} h_{\text{actual}}\geq h_{\text{mi}{\text{n}}_{-}\text{thresh}} \end{array}$$

Because the control variable uses the convention [X Y Z], the task references we compute have the following structure:

(19) $$\begin{array}{l} w{\dot{x}}_{max}=\left[\begin{array}{c} 0\\ 0\\ {\;}^{w}v_{z} \end{array}\right] \end{array}$$

(20) $$\begin{array}{l} w{\dot{x}}_{max}=k\left[\left(d_{limit}+\Delta \right){-}^{w}d_{sensor}\right] \end{array}$$

where *k* is the control gain, *d*_*limit*_ is the desired minimum distance from the seafloor, Δ is the safety distance at which the activation of the task starts to trigger, ${\;}^{w}d_{sensor\;}$ is the third component of the distance vector, measured by the sensor and projected on the world frame < w >.

Here, we provide a direction of the velocity we need to control the movement along the z-axis. We use the same vehicle position Jacobi matrix but only select the components associated with the z-axis. To ensure that UVMS achieves the goal of “MAC”, the activation variable *A*_*mac*_ for this task have the following structure:

(21) $$\begin{array}{l} A_{mac}=\left[\begin{array}{ccc} a_{1,1} & 0 & 0\\ 0 & a_{2,2} & 0\\ 0 & 0 & a_{3,3} \end{array}\right]=\left[\begin{array}{ccc} 0 & 0 & 0\\ 0 & 0 & 0\\ 0 & 0 & a_{3,3} \end{array}\right] \end{array}$$

where *a*_1,1_ and *a*_2,2_ are equal to zero because the intent is to constrain the system for only the velocity in the *z* direction of the world frame < w >, given by Equation (21). Thus, for *x* and *y* components, the activation should always remain zero.

The desired behavior for reactive control of our inequality objective is:

1. The task should be fully active only when the inequality is false.
2. The transition for activation should be smooth.

Therefore, we use *DecreasingBellshaped* function in order to calculate *a*_3,3_:

(22) $$\begin{array}{l} a_{3,3}≜\{\begin{array}{l} 1\;h_{actual\;}<h_{\min_{-}thresh}\\ decreasingbell\;h_{\min_{-}thresh}\leq h_{actual\;}\\ \leq h_{\min_{-}thresh}+\Delta \\ 0\;h_{actual\;}>h_{\min_{-}thresh}+\Delta \end{array} \end{array}$$

We compute variable ${\;}^{w}v_{z}$ using the below equation:

(23) $$\begin{array}{l} {\;}^{w}v_{z}=\lambda \left({\;}^{w}\bar{h}{-}^{w}h_{actual\;}\right) \end{array}$$

where ${\;}^{w}h_{actual\;}$ is the distance given by the ${\;}^{v}d_{sensor}$ on the z-axis of the world frame < w >. This quantity represents the vehicle's distance from the seafloor seen from the vehicle itself.

Note: ^*y*^*x*, where *y* represents the name of the frame and *x* represents the vector.

The division of the minimum altitude threshold defines the interval.

- *h*_*actual*_ < *h*_min_−_*thresh*_: The reference velocity (Equation 23) will be positive and will drive the robot toward *h*_min_−_*thresh*_ + Δ with activation *a*_3,3_ = 1.
- *h*_min_−_*thresh*_ < *h*_*actual*_ < *h*_min_−_*thresh*_ + Δ: The reference velocity (Equation 23) will be positive and will drive the robot toward *h*_min_−_*thresh*_ + Δ with activation *a*_3,3_ < 1 and *a*_3,3_ > 0 (transition region).
- *h*_*actual*_ > *h*_min_−_*thresh*_ + Δ: The reference velocity (Equation 23) will be negative, but the activation *a*_3,3_ = 0 and therefore does not have any effect on the UVMS.

As seen from the above intervals, we choose to implement a minimum altitude threshold $w\overset{\;}{\bar{h}}=h_{\min_{-}thresh}+\Delta$, which helps us avoid over-constraining the system.

Therefore, different thresholds apply to different types of seafloor. We have simulated different values of the minimum altitude in Table 2. These values all have the same k-gain; we summarize the possible scenarios.

**Table 2:** Comparison between behavior for different thresholds for different types of seafloor.

**Type of seafloor**	***h*_min_−_*thresh*_ = 1**	***h*_min_−_*thresh*_ = 5**	***h*_min_−_*thresh*_ = 10**
Almost flat	Safe	Safe	Safe
Small protuberances	Not completely safe	Safe	Safe
Large protuberances	Not safe	Not always safe	Safe

The *sensorDistance* we used is the distance measured by the sensor on the UVMS along the z-axis of the sensor frame < s >.

(24) $$\begin{array}{l} {\;}^{v}d_{sensor}=\left[\begin{array}{c} 0\\ 0\\ {\;}^{s}sensorDistance \end{array}\right] \end{array}$$

However, ${\;}^{v}d_{sensor}$ is the distance vector measured by the sensor and projected on the vehicle frame < v >. Since we need to project it onto the world frame < w >, we apply the following rotation matrix:

(25) $$\begin{array}{l} {\;}^{w}d_{sensor}{=}^{w}{R_{v}}^{v}d_{sensor} \end{array}$$

To obtain the distance between seafloor and robot in the world frame < w >, we use the below equation:

(26) $$\begin{array}{l} {\;}^{w}d_{sensor}=\left[\begin{array}{c} {\;}^{w}X-component\\ {\;}^{w}-component\\ {\;}^{w}h_{actural\;}\\ 0 \end{array}\right]{=}^{w}{T_{v}}^{v}d_{sensor} \end{array}$$

where with *h*_*actual*_ extracted by the z component of the sensorDistance projected in the world frame < w >. We assume that sensor frame < s > and vehicle frame < v > coincide.

#### The next highest priority “HA” and its mutually binding “PC” and “LA” tasks

The “HA” is the horizontal attitude task set in this paper and is the next highest priority task. This task ensures that UVMS does not flip for a given reference speed. Suppose we try to swap the priority of “PC” and “HA”. In that case, the vehicle will try to achieve the direction of the target if its horizontal roll or vertical sway is different from zero. Therefore, when we change the priority, the behavior observed during the simulation is almost the same as when the horizontal attitude task is disabled. The horizontal attitude is not enabled, and it will appear that the UVMS is not parallel to the bottom. In addition, swapping priorities is wrong because a horizontal attitude is a safety task and should have a higher priority than the task that defines the action. As can be seen above, the multiple solutions of the higher priority task (“HA”) already constrained task “PC”. We give that the vehicle position control task “PC” and the horizontal attitude task “HA” are mutually constrained as follows:

(27) $$\begin{array}{l} \dot{y}=\rho_{1}+Q_{1}{ż}_{1}\;\forall {ż}_{1} \end{array}$$

The vehicle position control task “PC” is set in this paper. We initialize the UVMS at a place far from the seabed, i.e., at sea level, and give the target position far enough, considering the actual situation. This task aims to perform the vehicle position control task to ensure that the vehicle achieves the required position and orientation.

The Jacobian of the task “PC” is:

(28) $$\begin{array}{l} {\;}^{w}{\dot{x}}_{2}{=}^{w}{\dot{x}}_{posc}=J_{posc}{\;}^{v}\dot{y} \end{array}$$

$$\begin{array}{l} {\dot{x}}_{posc}\in R^{6},J_{posc}\in R^{6\times 13},\;\dot{y}\in R^{13} \end{array}$$

where the Jacobi matrix has 13 columns corresponding to the dimensions of the control variables at the kinematic level, the control variables are the seven joints of the manipulator and the six D.O.F of the vehicle base. Six rows correspond to the dimensions of the reference velocity. The time volume is the difference between the initial and target positions or λ-value.

(29) $$\begin{array}{l} {\;}^{w}{\dot{x}}_{posc6\times 1}=\left[\begin{array}{c} {\;}^{w}v_{3\times 1}\\ {\;}^{w}\omega_{3\times 1} \end{array}\right]=\left[\begin{array}{ccc} 0_{3\times 7} & {\;}^{w}R_{v3\times 3} & 0_{3\times 3}\\ 0_{3\times 7} & 0_{3\times 3} & {\;}^{w}R_{v}3\times 3 \end{array}\right]\left[\begin{array}{c} {\dot{q}}_{7\times 1}\\ {\;}^{v}v_{3\times 1}\\ {\;}^{v}\omega_{3\times 1} \end{array}\right] \end{array}$$

Since the task “PC” is a reactive control task, the task reference is computed using the formula of the closed-loop feedback reference rate such that:

- Required position:

(30) $$\begin{array}{l} {\;}^{w}\bar{\nu }=\lambda_{l}\left({\;}^{w}{\bar{x}}_{position\text{\_}goal}{-}^{w}x_{actual\text{\_}position}\right) \end{array}$$

- Required orientation:

(31) $$\begin{array}{l} {\;}^{w}\bar{\omega }=\lambda_{a}VersorLemma\;\left({\;}^{w}{\bar{x}}_{orientation\text{\_}goal}{,}^{w}x_{actual\text{\_}orientation}\right) \end{array}$$

and in a compact form:


(32) $$\begin{array}{l} w\bar{x}=\left[\begin{array}{cc} \lambda_{1} & \lambda_{r} \end{array}\right]\left[\begin{array}{cc} w & r\\ {\;}^{w}\delta & \theta \end{array}\right] \end{array}$$

with:


(33) $$\begin{array}{l} \left[\begin{array}{c} r\\ \theta \end{array}\right]=CartError\left({\;}^{w}{\bar{x}}_{position\text{\_}goal}{,}^{w}x_{actual\text{\_}position}\right) \end{array}$$

We use “*CartError*” function to calculate *r* and θ, and we set the two gains equal to λ_*l*_ = 0.2 and λ_*a*_ = 0.5.

The altitude control task “LA” is set in this paper, and since “LA” is an action definition (AD) target, it is placed after the security task. It is important to note that the objectives of “LA” and “PC” are so different that it is unlikely that they will be activated simultaneously. Therefore, their relative priority does not affect the solution. There are two main differences between this task and the minimum altitude control task in this paper, the first being that landing is not a safety task but rather an action that defines a safety task. While using the minimum altitude task to avoid collisions with the seafloor, the landing task “LA” defines an action as a vehicle position, therefore, has a lower priority; the second difference is that the minimum altitude is unequal to the landing is an equal task.

The Jacobian for task “LA” is:

(34) $${\;}^{w}{\dot{x}}_{la}{=}^{w}v_{3\times 1}=\;\left[\begin{array}{ccc} 0_{3\times 7} & {\;}^{w}R_{v}{\;}_{3\times 3} & 0_{3\times 3} \end{array}\right]\;\left[\begin{array}{c} {\dot{q}}_{7\times 1}\\ v_{3\times 1}\\ {\;}^{v}\omega_{3\times 1} \end{array}\right]$$

As with the minimum altitude control objective, only the Jacobi matrix is needed to control the components along the z-axis. We calculate the task reference as:

(35) $$\begin{array}{l} {\;}^{w}{\dot{\bar{x}}}_{\text{land}}=k\left[\left(d_{\text{landing}}+\Delta_{\text{safeguard}}\right){-}^{w}d_{\text{sensor}}\right] \end{array}$$

where *k* is the control gain, *d*_landing_ is the distance from the seafloor, in this case, Δ_safeguard_ is set to 0.17 m to avoid interpenetration between UVMS and the seafloor. ${\;}^{w}d_{\text{sensor}}$ is the component along the z axis of the distance vector measured by the sensor and projected on the world frame < w >.

#### Goal control alignment target task “AT”

If we only use the position control task “PC”, we can only guarantee that we reach the target position, but not that we are aligned with the job target to complete the job task. We must add additional constraints to make the vehicle face the target task. The approach we take is to add an alignment task between the vehicle x-axis and the target. In particular, the vehicle x-axis should be aligned with the projection of the unit vector on the inertial level that connects the vehicle frame < v > to the target frame < o >.

So we add the target control alignment task “AT” to the task hierarchy. We decide to place “AT” under “PC” to take advantage of the remaining arbitrariness to align the robot in the direction we want during the target task activation phase. Before landing, the robot tries to align itself with the operational target. When it reaches sufficient alignment (θ < 0.5), the “PC” phase is activated, during which the robot reaches the target position and aligns as much as possible.

The main goal of this task is to align the vehicle, especially the direction to the target. For this purpose, we have to calculate the rotation vector ρ between the two necessary vectors.

The two vectors are:

- *a*: x-axis of the vehicle frame

(36) $$\begin{array}{l} {\;}^{v}a=\left[\begin{array}{c} 1\\ 0\\ 0 \end{array}\right] \end{array}$$

- *b*: A vector between the vehicle frame and target projected onto the inertial horizontal plane and expressed in the vehicle frame < v >.

(37) $$\begin{array}{l} {\;}^{v}b{=}^{v}R_{w}{\left(I-kk^{⊤}\right)}^{w}Distance\text{\_}target \end{array}$$

Now, we can proceed to compute ρ vector in the following way:

(38) $$\begin{array}{l} a\wedge b=n\sin\left(\theta \right) \end{array}$$

(39) $$\begin{array}{l} \rho =n\theta \end{array}$$

where that returns the direction *n* and the magnitude θ that vector *a* must perform to be aligned with *b*. Since our final goal is to have the x-axis of the vehicle aligned with the target, we have to study the behavior of this resulting vector during the time. Thus, we want:

(40) $$\begin{array}{l} {\overset{.}{x}}_{ref}=\overset{.}{\rho }=\gamma n\theta \end{array}$$

Considering a generic observer, we have:

(41) $$\begin{array}{l} D_{\alpha }\rho =\dot{\theta }n+\theta D_{a}\left(n\right)=nn\omega_{b/\alpha }+N_{\alpha }\left(\theta \right)\left(\omega_{b/\alpha },\omega_{a/\alpha }\right) \end{array}$$

Considering an observer inside the rigid space of the vehicle frame < v > on which there is vector *a*:

(42) $$\begin{array}{l} D_{a}\rho =nn\omega_{b/a}+N_{\alpha }\left(\theta \right)\omega_{b/a}=nn\omega_{b/a} \end{array}$$

where the second term is exactly equal to zero due to the fact that we have ρ and $\overset{.}{\rho }$ aligned. Finally, we want:

(43) $$\begin{array}{l} \gamma n\theta =\omega_{b/a} \end{array}$$

Since the quantity ω_b/a_ is not easy to compute we can compute it using the law of addition of angular velocity vectors:

(44) $$\begin{array}{l} \omega_{\text{b/a}}=\omega_{\text{b/w}}\text{-}\omega_{\text{a/w}} \end{array}$$

where ω_a/w_ is the angular velocity of the vehicle with respect to the world. ω_b/w_ is the angular velocity given by the movement of the vehicle with respect to the target that produces a change of direction of unit vector joining the vehicle frame < v > to the target frame < o >. It is important to compute this quantities using the same observer as for ρ, computed in Equation 38, thus in our case:

It is essential to calculate this quantity using the same observer as for ρ in Equation (38), therefore, in the vehicle frame < v >.

(45) $$\begin{array}{l} {\;}^{v}\omega_{\text{b/a}}{=}^{v}\omega_{\text{b/w}}{-}^{v}\omega_{\text{a/w}} \end{array}$$

in particular:

(46) $$\begin{array}{l} {\;}^{v}\omega_{\text{b/a}}=\left(\frac{{\;}^{v}b}{\left|{|}^{v}b|\right|}\wedge \frac{{-}^{v}vp}{\left|{|}^{v}vp|\right|}\right)\frac{\left|{|}^{v}vp|\right|}{\left|{|}^{v}b|\right|} \end{array}$$

where ^*v*^*vp* is the projection of the linear velocity of the vehicle on the inertial horizontal plane expressed in vehicle frame < v > thus:

(47) $$\begin{array}{l} {\;}^{v}vp{=}^{v}R_{w}{\left(I-kk^{T}\right)}^{w}{R_{v}}^{v}v \end{array}$$

We can now proceed to compute the desired Jacobian matrix that, according to Equations 5 and 6, must be the following one:

(48) $$\dot{x}=\dot{\rho }=\left[0_{3\times 7}-\frac{\left\|{\;}^{v}vp\right\|}{\left\|{\;}^{v}b\right\|}\left(\frac{{\;}^{v}b}{\left\|{\;}^{v}b\right\|}\wedge \frac{{\;}^{v}R_{w}{\left(I-kk^{T}\right)}^{w}R_{v}}{\left\|{\;}^{v}vp\right\|}\right)1\right]\left[\begin{array}{c} \dot{q}\\ {\;}^{v}v\\ {\;}^{v}w \end{array}\right]$$

Moreover, the Jacobian relationship for the alignment target control task “AT” is derived from the following formula.

(49) $$\begin{array}{l} D_{w}\left(p\right)=J_{at}\dot{y} \end{array}$$

where *p* is the misalignment vector. *D*_*w*_(*p*) is the derivative of the misalignment vector. *J*_*at*_ is the Jacobian we want to compute. We know that

(50) $$\begin{array}{l} D_{w}\left(p\right){=}^{w}{\text{n}}_{\rho }\dot{\theta }+\underset{\text{orthogonal}}{\underset{︸}{\theta {D_{w}}^{w}{\text{n}}_{\rho }}} \end{array}$$

Since we are not interested in the orthogonal components, we can ignore them. By looking at the first term of the sum, we know that ${\;}^{w}{\text{n}}_{\rho }$ is the misalignment vector projected on the world and θ can be written as

(51) $$\begin{array}{l} \dot{\theta }{=}^{w}\omega_{v}{-}^{w}\omega_{\text{goal}} \end{array}$$

where ${\;}^{w}\omega_{v}$ is the angular velocity of the vehicle, referred to the world. ${\;}^{w}\omega_{\text{goal}}$ is the angular velocity of the projected distance between the target and the vehicle, referred to the world.

By describing ${\;}^{w}\omega_{v}$ in terms of $\dot{y}$, we can deduce the following Jacobian

(52) $$\begin{array}{l} {\text{J}}_{vehicle}=\left[\begin{array}{cc} 0_{3\times 10} & {\;}^{w}{R_{v}}_{3\times 3} \end{array}\right] \end{array}$$

In order to describe ${\;}^{w}\omega_{\text{goal}}$ in terms of $\dot{y}$, we use the following relationship

(53) $$\begin{array}{l} {\;}^{w}\upsilon_{v}{=}^{w}\omega_{\text{goal}}\wedge^{w}d \end{array}$$

where ${\;}^{w}\upsilon_{v}$ is the linear velocity of the vehicle projected on the world frame < w >.^*w*^*d* is the projected distance on the horizontal inertial frame, between the target and the z-axis of the vehicle.

(54) $$\begin{array}{l} J_{goal}=\frac{1}{\left|{|}^{w}d^{2}|\right|}\left[{\;}^{w}d\wedge \right]\begin{array}{c} {\;}^{w}\upsilon_{v} \end{array} \end{array}$$

Since we are interested only in the *x* and *y* component of ${\;}^{w}\upsilon_{v}$, we select such components by premultiplying as given below:

(55) $$\begin{array}{l} J_{goal}=\frac{1}{\left|{|}^{w}d^{2}|\right|}{\left[{\;}^{w}d\wedge \right]}^{\;}\left[\begin{array}{ccc} 1 & 0 & 0\\ 0 & 1 & 0\\ 0 & 0 & 0 \end{array}\right]\begin{array}{c} {\;}^{w}\upsilon_{v} \end{array} \end{array}$$

From the last equation, we deduce the following Jacobian

(56) $$\begin{array}{l} J_{goal}=\frac{1}{\left|{|}^{w}d^{2}|\right|}\left[{\;}^{w}d\wedge \right]\left[\begin{array}{ccc} 1 & 0 & 0\\ 0 & 1 & 0\\ 0 & 0 & 0 \end{array}\right]\left[\begin{array}{ccc} 0_{3\times 7} & {\;}^{w}{R_{v}}_{3\times 3} & 0_{3\times 3} \end{array}\right] \end{array}$$

The resulting Jacobian is obtained by substitution and it is equal to

(57) $$\begin{array}{l} J_{at}{=}^{w}n_{r}^{⊤}\left[J_{vehicle}-J_{goal}\right] \end{array}$$

We compute the task reference as:

(58) $$\begin{array}{l} {\;}^{w}{\dot{\bar{x}}}_{\text{at}}=k\left(0-|{|}^{w}p||\right) \end{array}$$

where *k* is the control gain. ||^*w*^*p*|| is the norm of the misalignment vector, in this case, we want it to be 0.

## Trajectory planning

### Trajectory optimization goal

Once the task is divided into subtasks, the placement of the job manipulator is critical because it affects the subsequent manipulation tasks. Poor essential placement may even fail to reach the final target state. A significant problem with this approach is the suboptimality of the generated solution trajectories. Even though optimal solutions can be generated for each subtask, the set of these solutions does not necessarily produce a globally optimal solution. The goal state of the previous subtask will significantly affect the planning of the next task. It may even prevent the generation of feasible solutions, resulting in the need to replan the previous task. As a result, this approach will lead to local optima, global suboptimal paths, or many unsuccessful motion planning queries. Combining the system with high-degree-of-freedom maneuver planning for the entire task can alleviate the suboptimal global problem, but this requires extensive computation. Motion coordination between the vehicle and the manipulator, collision checking and self-collision checking with the environment, and motion constraints are some added complexities in this approach.

In this research, the trajectory optimization objective is the lowest priority to address this issue. After the vehicle has completed all priority tasks, we focus on considering the desired trajectory of the end-effector on the UVMS. We convert the trajectory planning problem into finding feasible joint trajectories considering the priority tasks first while optimizing the cost function given by the expectation. In velocity-resolved inverse kinematics, the task is the expectation of the robot configuration function, represented in the task description by an equation or inequality constraint. Finally, the trajectory planning problem for the end-effector can be formulated as the following optimization problem.

The optimized smooth trajectory needs to consider its boundary conditions, including the start and end states, the relay node as the waypoint through which the robot passes, and the smoothing criterion to evaluate whether the generated trajectory is smooth. Knowing the angles to be reached by M joint, a polynomial fit will result in segment M-1 trajectories, each represented by a polynomial, and the set of trajectories needs to satisfy the following constraints:

- Desired angle constraint:

(59) $$\begin{array}{l} \{\begin{array}{cc} f_{j}^{\left(k\right)}\left(T_{j-1}\right) & =x_{0,j}^{\left(k\right)}\\ f_{j}^{\left(k\right)}\left(T_{j}\right) & =x_{T,j}^{\left(k\right)} \end{array} \end{array}$$

- Continuity constraint:

The velocity and acceleration of adjacent trajectories are continuous:

(60) $$\begin{array}{l} f_{j}^{\left(k\right)}\left(T_{j}\right)=f_{j+1}^{\left(k\right)}\left(T_{j}\right) \end{array}$$

The cost function is chosen to minimize the Snap value for all trajectories. Snap is the fourth-order derivative of position, and minimizing Snap allows the end-effector to meet the autonomous operational movement suitable for UVMS. At the same time, its kinetic states, such as velocity and acceleration, cannot change abruptly. Reducing the range of acceleration and deceleration enables UVMS to work longer in energy-limited underwater environments.

The cost function determined to minimize snap is expressed as follows:

(61) $$\begin{array}{l} J\left(T\right)=\int_{T_{j-1}}^{T_{j}}{\left(f^{4}\left(t\right)\right)}^{2}dt\\ \;=\underset{i\geq 4,l\geq 4}{\sum }\frac{i\left(i-1\right)\left(i-2\right)\left(i-3\right)j\left(l-1\right)\left(l-2\right)\left(l-3\right)}{i+l-7}\\ \;\times \left(T_{j}^{i+l-7}-T_{j-1}^{i+l-7}\right)p_{i}p_{j} \end{array}$$

The coefficients of each order are extracted separately, and the cost function can be written in the quadratic form:

(62) $$\begin{array}{l} J_{j}\left(T\right)={\text{p}}_{j}^{T}{\text{Q}}_{j}{\text{p}}_{j} \end{array}$$

where **Q**_*j*_ is the Hessian matrix that transforms the trajectory optimization problem into a quadratic programming problem, for the final trajectory of the manipulator, each trajectory point should satisfy the following constraints.

Track point constraint, each trajectory should pass through the track point obtained by the path search, and the displacement, speed, acceleration, jerk, and snap at the track point should all exist. To satisfy $f_{\mu }^{\left(k\right)}\left(T_{i}\right)=d_{ik}$, where μ ∈ {*x, y, z*}, *k* ∈ {0, 1, 2, 3}, *i* ∈ {1, 2, ⋯ , *M*} continuity constraints, the displacement, velocity, acceleration, jerk, and snap at the track point should also be continuous. That is satisfied $f_{\mu }^{\left(k\right)}\left(T_{i}\right)=d_{ik}$, where μ ∈ {*x, y, z*}, *k* ∈ {0, 1, 2, 3}, *i* ∈ {1, 2, ⋯ , *M*}. It can be written as an equality constraint.

We need to fix each trajectory time *T*_*j*_ = 0.1*s* for all joints. Ensure that all robot joints reach the desired angle and end position at the same moment is always stable.

Summarizing the above constraints and cost functions, they are written in matrix form.

- Desired angle constraint:

(63) $$\begin{array}{l} f_{j}^{\left(k\right)}\left(T_{j}\right)=x_{j}^{\left(k\right)} \end{array}$$

(64) $$\begin{array}{l} \Rightarrow \underset{i\geq k}{\sum }\frac{i!}{\left(i-k\right)!}T_{j}^{i-k}p_{j,i}=x_{T,j}^{\left(k\right)} \end{array}$$

(65) $$\begin{array}{l} \Rightarrow \left[...\begin{array}{ccc} \cdots \; & \frac{i!}{\left(i-k\right)!}T_{j}^{i-k}\cdots \; & ... \end{array}\right]\left[\begin{array}{c} :\\ p_{j,i}\\ : \end{array}\right]=x_{T,j}^{\left(k\right)} \end{array}$$

(66) $$\begin{array}{l} \Rightarrow \left[\begin{array}{c} \frac{i!}{\left(i-k\right)!}T_{j-1}^{i-k}\\ :\\ :\\ \frac{i!}{\left(i-k\right)!}T_{j}^{i-k} \end{array}\right]\left[\begin{array}{c} :\\ p_{j,i}\\ : \end{array}\right]=\left[\begin{array}{c} x_{0,j}^{\left(k\right)}\\ x_{T,j}^{\left(k\right)} \end{array}\right] \end{array}$$

(67) $$\begin{array}{l} \Rightarrow {\text{A}}_{j}{\text{p}}_{j}={\text{d}}_{j} \end{array}$$

- Continuity constraint: smoothness constraint ensures continuity between trajectory segments without giving a specific derivative.

(68) $$\begin{array}{l} f_{j}^{\left(k\right)}\left(T_{j}\right)=f_{j+1}^{\left(k\right)}\left(T_{j}\right) \end{array}$$

(69) $$\begin{array}{l} \Rightarrow \underset{i\geq k}{\sum }\frac{i!}{\left(i-k\right)!}T_{j}^{i-k}p_{j,i}-\underset{l\geq k}{\sum }\frac{l!}{\left(l-k\right)!}T_{j}^{l-k}p_{j+1,l}=0 \end{array}$$

(70) $$\begin{array}{l} \Rightarrow \left[\begin{array}{cccccc} \cdots \; & \frac{i!}{\left(i-k\right)!}T_{j}^{i-k}\; & ...\; & ...\; & -\frac{l!}{\left(l-k\right)!}T_{j}^{l-k}\; & \cdots \end{array}\right]\\ \;\left[\begin{array}{c} p_{j,i}\\ :\\ :\\ p_{j+1,l} \end{array}\right]=0 \end{array}$$

(71) $$\begin{array}{l} \Rightarrow \left[{\text{A}}_{j}-{\text{A}}_{j+1}\right]\left[\begin{array}{c} {\text{p}}_{j}\\ {\text{p}}_{j+1} \end{array}\right]=0 \end{array}$$

Substitution function:


(72) $$\begin{array}{l} \begin{array}{c} J\left(T\right)=\int_{T_{j-1}}^{T_{j}}{\left(f^{4}\left(t\right)\right)}^{2}dt\\ ={\left[\begin{array}{c} :\\ p_{i}\\ : \end{array}\right]}^{T}\left[\ldots \frac{i\left(i-1\right)\left(i-2\right)\left(i-3\right)l\left(l-1\right)\left(l-2\right)\left(l-3\right)}{i+l-7}T^{i+l-7}\ldots \right]\left[\begin{array}{c} :\\ p_{l}\\ : \end{array}\right] \end{array} \end{array}$$

(73) $$\begin{array}{l} J_{j}\left(T\right)={\text{p}}_{j}^{T}{\text{Q}}_{j}{\text{p}}_{j} \end{array}$$

Writing the above problem as an equation constraint in standard form, then the quadratic programming problem can be expressed as:


(74) $$\begin{array}{l} \min{\left[\begin{array}{c} {\text{p}}_{1}\\ :\\ {\text{p}}_{M} \end{array}\right]}^{T}\left[\begin{array}{ccc} {\text{Q}}_{1} & 0 & 0\\ 0 & : & 0\\ 0 & 0 & {\text{Q}}_{M} \end{array}\right]\left[\begin{array}{c} {\text{p}}_{1}\\ :\\ {\text{p}}_{M} \end{array}\right] \end{array}$$

(75) $$\begin{array}{l} s.t.\;{\text{A}}_{\text{eq}}\left[\begin{array}{c} {\text{p}}_{1}\\ :\\ {\text{p}}_{M} \end{array}\right]={\text{d}}_{eq} \end{array}$$

The above equation is a linear constraint quadratic programming problem (QP).

### Collision avoidance

UVMS requires only six degrees of freedom to reach an arbitrary position in underwater motion. Adding a manipulator gives the entire system more than six degrees of freedom, resulting in the redundancy of degrees of freedom. Kinematic redundancy allows the planner to satisfy additional constraints, such as collision avoidance. Researchers have mainly focused on approximating the robot or the obstacle with strictly convex targets and considering only the closest points in the detection algorithm to avoid collisions to reduce computational effort.

The optimization process has no environmental constraints after the trajectory planning solution based on the minimum snap principle. When a new trajectory is encountered after optimization, the obstacles force the trajectory to be modified again, wasting computational resources and reducing the planning frequency. For example, it is necessary to add constraints on the environment during optimization, generally based on hard constraint solving. The hard constraint solution is to generate a safe region in the environment by extending the algorithm and using it as a hard constraint. Adding hard constraints in the optimization process forms a convex polygon, which transforms the QP problem into a convex optimization problem that can be solved by convex optimization algorithms such as the interior point method.

While the process of underwater obstacle avoidance, most of the surrounding objects are non-strictly convex polyhedra, and these approximation methods are not accurate enough when operating in close range. The problems in the practical application process are ignored. Because the remaining safe regions are treated equally during optimization, there is no good way to handle the extreme cases with underwater sensor noise. The optimized trajectory may go past the edge of the safety zone. Once the controller makes an error, it leads to a severe failure of the manipulator body by colliding with the internal and external environment. Inspired by the penalty function, we propose a more intuitive collision-free motion planning method oriented to UVMS.

### An improved collision avoidance method based on soft constraint

By design, we use the principle of soft constraint to improve the collision avoidance method. The essence of the soft constraint method is to apply a “pushing force” to push the trajectory away from the direction of the obstacle. The core problem is the designed objective function. When the objective function is not set correctly, the path may hit an obstacle, which is the shortcoming of soft constraint. Therefore, a gradient-based optimization algorithm sets the objective function to impose a soft constraint on the underwater manipulator to push the underwater manipulator body away from the obstacle.

For Equation 16, the objective function becomes:

(76) $$\begin{array}{l} J=J_{s}+J_{c}+J_{d}=\lambda_{1}J_{1}+\lambda_{2}J_{2}+\lambda_{3}J_{3} \end{array}$$

(77) $$\begin{array}{l} J_{S}=\underset{\mu \in \left\{x,y,z\right\}}{\sum }\int_{0}^{T}\left(\frac{d^{k}f_{\mu }\left(t\right)}{dt^{k}}\right)dt \end{array}$$

(78) $$\begin{array}{l} {\left[\begin{array}{c} d_{F}\\ d_{P} \end{array}\right]}^{T}C^{T}M^{-T}QM^{-T}C\left[\begin{array}{c} d_{F}\\ d_{P} \end{array}\right]\\ ={\left[\begin{array}{c} d_{F}\\ d_{P} \end{array}\right]}^{T}\left[\begin{array}{cc} R_{FF} & R_{FP}\\ R_{PF} & R_{PP} \end{array}\right]\left[\begin{array}{c} d_{F}\\ d_{P} \end{array}\right] \end{array}$$

where the smoothness cost function *J*_*s*_ is the cost of smoothness generated using minimum-snap.

(79) $$\begin{array}{l} J_{c}=\int_{T_{0}}^{T_{M}}c\left(p\left(t\right)\right)ds\\ \;=\overset{T|\delta t}{\underset{k=0}{\sum }}c\left(p\left(T_{k}\right)\right)\|\|v\left(t\right)\|\|\delta t,T_{k}=T_{0}+k\delta t \end{array}$$

where the collision cost function *J*_*c*_, i.e., the collision cost, penalizes obstacles that are too close.

where the kinetic cost function *J*_*d*_ penalizes exceeding the kinetic constraints. Since the objective function of penalizing the velocity and acceleration is not a convex function, it needs to be solved by step-by-step derivation. The smooth term solution is shown in the previous derivation, and the relationship between the collision term and the free variables *d*_*pμ*_ is as follows.

(80) $$\begin{array}{l} J_{c}=\int \left\{\overset{T|\delta t}{\underset{k=0}{\sum }}\left\{\forall_{\mu }c\left(p\left(T_{k}\right)\right)||||v||||F+c\left(p\left(T_{k}\right)\right)\frac{v_{\mu }}{\left||||v|||\right|}G\right\}\delta td_{p\mu }\right\},\\ \;\mu \in \left\{x,y,z\right\} \end{array}$$

where the *F* and *G* are, respectively:

(81) $$\begin{array}{l} F=TL_{dp},\;G=TV_{m}L_{dp} \end{array}$$

where *L*_*dp*_ is the right half of the matrix *M*^−1^*C*, *V*_*m*_ is the mapping matrix of joint position variables to joint velocity variables, $T=\left[T_{k}^{0},T_{k}^{1},\ldots ,T_{k}^{n}\right]$.

The second-order derivative results in:

(82) $$\begin{array}{c} {\text{H}}_{o}=\left[\frac{\partial^{2}f_{o}}{\partial {\text{d}}_{P_{x}}^{2}},\frac{\partial^{2}f_{o}}{\partial {\text{d}}_{P_{y}^{2}}},\frac{\partial^{2}f_{o}}{\partial {\text{d}}_{P_{z}^{2}}}\right]\\ \frac{\partial^{2}f_{o}}{\partial {\text{d}}_{P\mu }^{2}}=\overset{\tau /\delta t}{\underset{k=0}{\sum }}\{{\text{F}}^{T}\nabla_{\mu }c\left(p\left(T_{k}\right)\right)\frac{v_{\mu }}{\left||v|\right|}\text{G}+{\text{F}}^{T}\nabla_{\mu }^{2}c\left(p\left(T_{k}\right)\right)||v||\text{F}\\ +{\text{G}}^{T}\nabla_{\mu }c\left(p\left(T_{k}\right)\right)\frac{v_{\mu }}{\left||v|\right|}\text{F}+{\text{G}}^{T}c\left(p\left(T_{k}\right)\right)\frac{v_{\mu }^{2}}{||v|{|}^{3}}\text{G}\}\delta t \end{array}$$

## Simulation results

In this section, the task priority processing strategy and soft-constrained trajectory optimization objective designed in this paper are verified on a kinematically redundant underwater vehicle manipulator system. The system consists of a free-floating underwater vehicle and a seven-function manipulator. The simulation is performed in a MATLAB/Simulink environment.

### Case 1: Test the given tasks

The simulation phase first initializes the UVMS for safe waypoint navigation. Afterward, we move the vehicle to a position close to the currently defined end-effector target position, slightly above the target position. Finally, the action change is triggered, and the UVMS executes the ocean float operation. We do not consider any disturbances and assume that the robot can provide the desired speed without delays. In addition, once the robot reaches the desired position, the rest of the tasks are responsible for the end-effector reaching the desired target position and orientation, so the “PC” task will also be closed. For the end-effector to operate as a stationary-based robot, we need to constrain the vehicle not to move. Because, as we noticed, the vehicle will “help” the arm to reach the desired position by moving itself (in line with the expected behavior of the tool task). To avoid this problem, we need to perform a non-reactive task to constrain the vehicle to move or reach the operating position where the task will make the vehicle move. In this case, we test UVMS landing on the seafloor, try the vehicle to its target coordinate system, and then use the end-effector to reach the operational target position. Observe whether the vehicle does not move and perform the ocean float operation of the UVMS.

The uniform hierarchy of tasks we use and their priorities, with the addition of constrained tasks at the priority level, is described in Table 3, with non-reactive tasks (“NR”) added at the top of the hierarchy to constrain the vehicle not to move.

**Table 3:** Control tasks and priorities for fixed base manipulation operations.

**Priority**	**Category**	**Description**	**Objective**
1	Constraint	[NR,E,C]	NR
2	Safety	[R,I,S]	MAC
3	Prerequisite	[R,I,P]	HA
4	Action-defining	[R,E,AD]	LA
5	Prerequisite	[R,E,P]	AT
6	Action-defining	[R,E,AD]	PC

[R/NR, I/E, C/S/P/AD] Name of the task/objective.

We have the following tasks in an active/inactive state for each of the different phases, is described in Table 4.

**Table 4:** Examples of external activation states for different tasks.

**Priority**	**Tasks**	**Way point**	**Alignment**	**Landing**	**Tool frame**
1	NR	0	0	0	1
2	MAC	1	1	0	0
3	HA	1	1	1	1
4	LA	0	0	1	0
5	AT	0	1	1	0
6	PC	1	0	0	0

0/1 External inactive/active.

We conducted a multitask prioritization strategy experiment to explain the task prioritization strategy better. First, multitasking is divided into multiple action phases.

**Figure 5:**
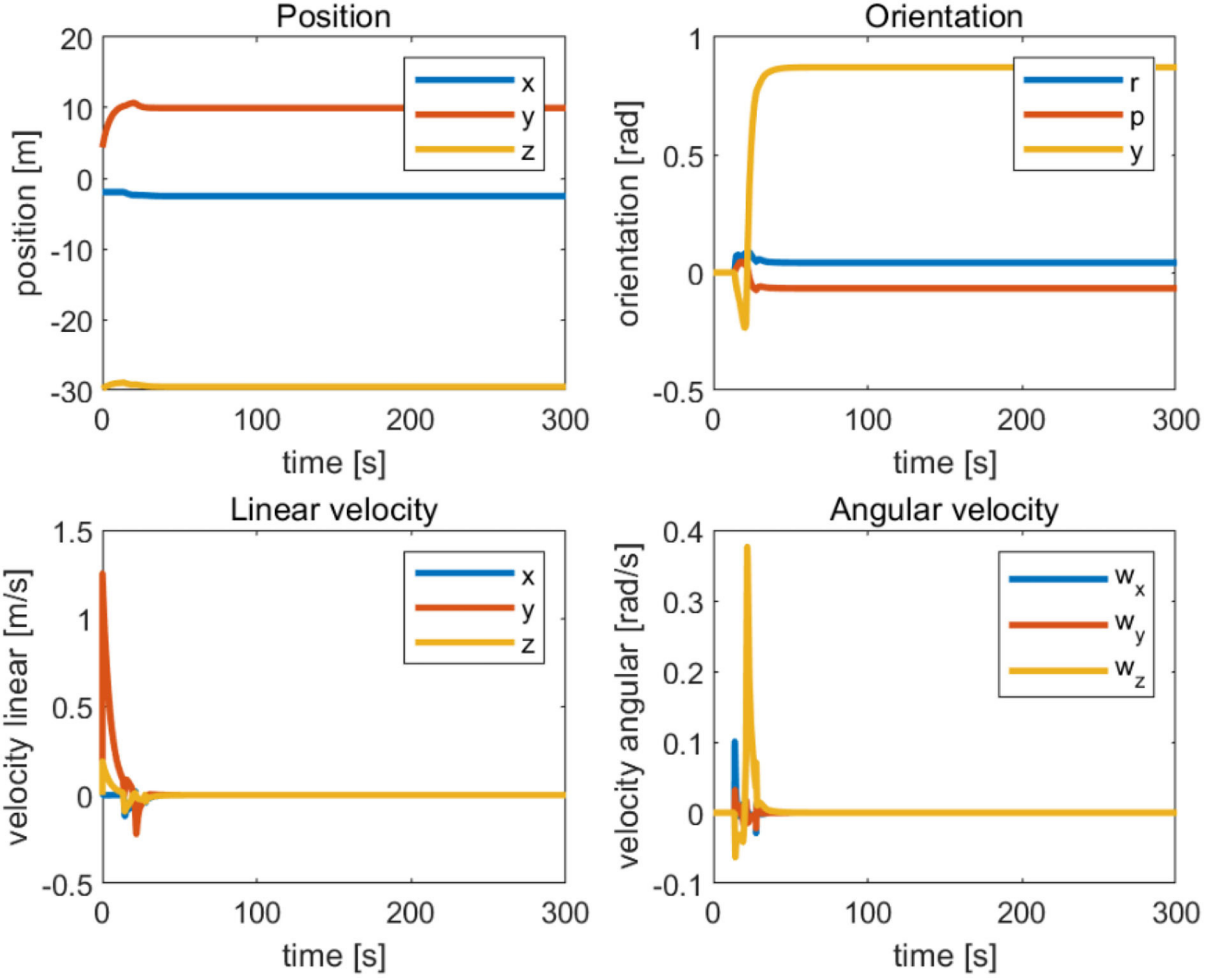
Position-directional linear velocity angular velocity tracking curve of the completed vehicle initialization.

1. Action A, safe waypoint navigation with all the safety tasks enabled. This action finishes when the position error is below a fixed threshold (in this case 0.1 m), as Figure 5 shows.
2. Action B, alignment to the nodule with all the safety tasks enabled. This action finishes when the misalignment error is below a fixed threshold (in this case 0.07 m).
3. Action C, landing, and smooth rotation align with the target. This action finishes when the vehicle touches the seafloor (in the simulation, this happens at approximately 0.17 m).
4. Action D, manipulator actuation after landing. In this action, Vehicle Null Velocity task is enabled, preventing vehicle movements. The only movement will be the extension of the manipulator to reach the desired target position.

**Figure 6:**
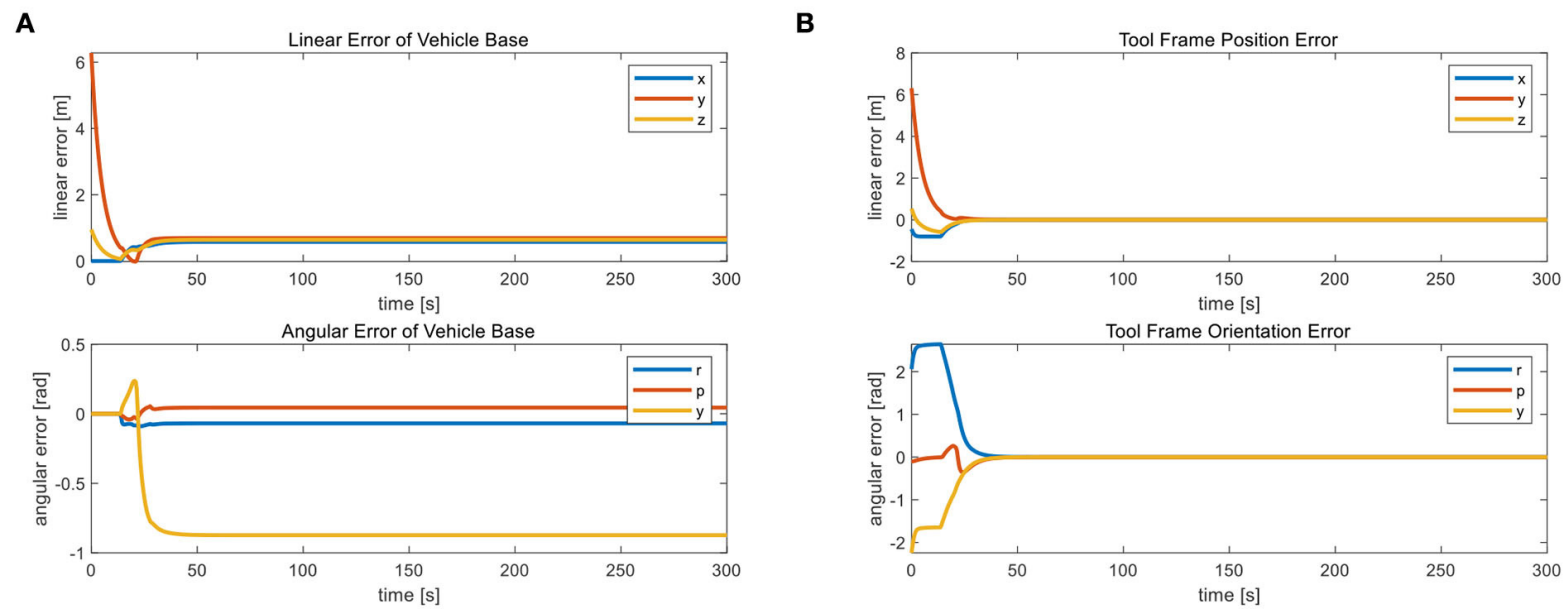
**(A)** The change curve of vehicle base error during the test and **(B)** the tool frame error.

**Figure 7:**
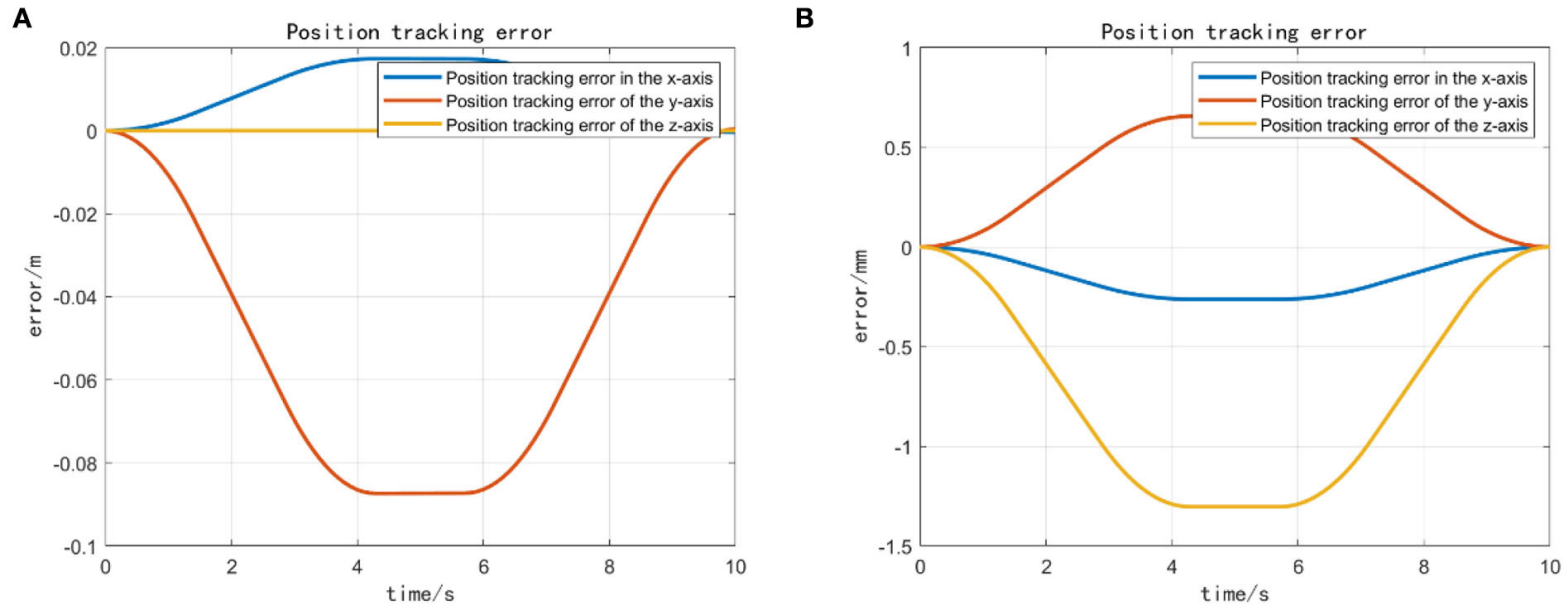
**(A)** The vehicle error completed for action A and **(B)** the unaligned error vehicle error completed for action B.

**Figure 8:**
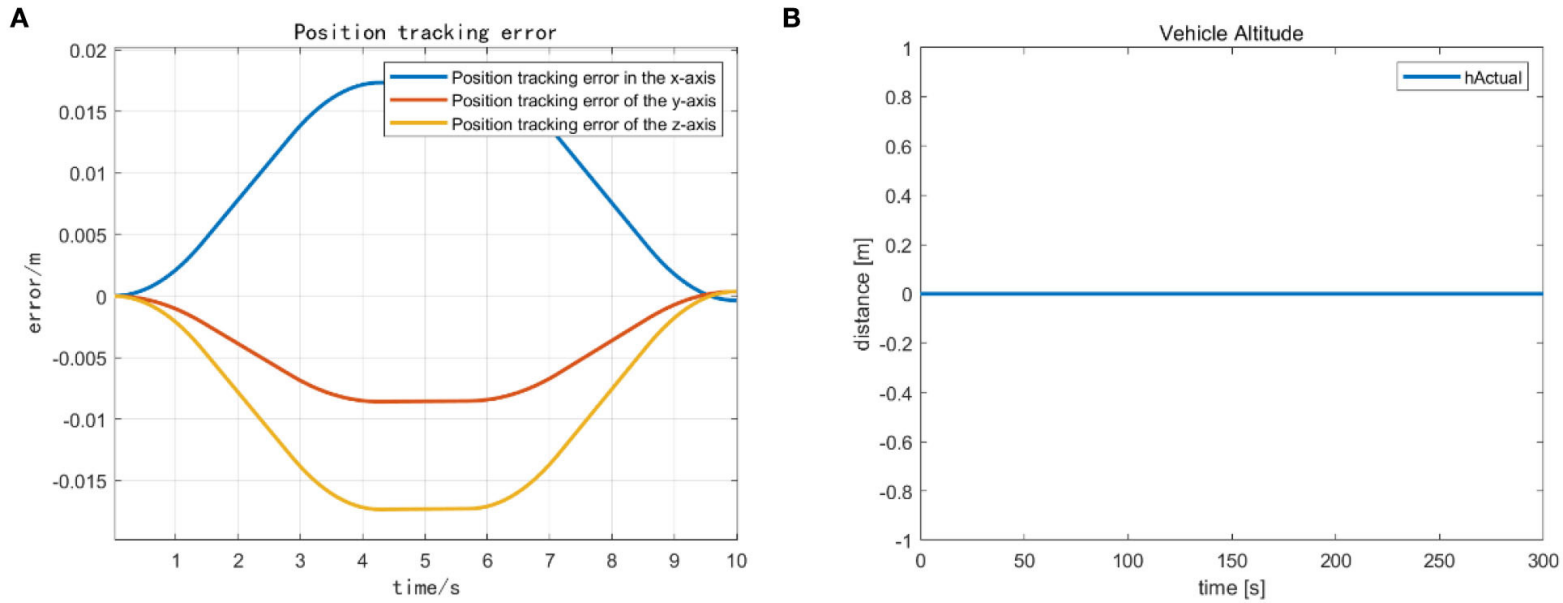
**(A)** The vehicle error for the completion of action C. **(B)** The smooth landing state of the vehicle after action C.

From Figure 6, we observe that the position and orientation errors of the vehicle base remain almost constant after 30 s of simulation when the UVMS has completed the landing phase and started the tool holder phase. Therefore, the tool frame phase's active task “NR” helps us achieve a fixed datum operation. Because after a reasonable time, the position error *r* and orientation error θ converge to near zero. When the error of the carriage position remains constant, the tool frame error of the manipulator operation is almost zero. Action A's fixed threshold of position error is within 0.1 m. Action B sets the fixed maximum of unaligned error by 1.3 mm, well below the set fixed threshold of 0.07 m, which provides the basis for the subsequent accurate completion of the operational target. Figure 7 shows the corresponding simulation results. Figure 8 shows that the maximum range of vehicle error aligned with the target is 0.17 m in Action C. The Action ends when the vehicle touches the seabed, i.e., when the height is 0.

Since the Vehicle Null only puts vehicle velocities to zero, it cannot compensate for the disturbance. In a similar scenario, the currents will influence the UVMS: the vehicle can arrive at the desired position and land if the disturbance is not too big. However, when the vehicle Null task is triggered in Action D, the UVMS will drift, eventually losing its target position. The manipulator will keep trying to reach the goal by stretching as much as possible. With a check on the position error during the mission phase update, it is possible to return to Action A and achieve the desired position again. Then the task phase update transition requires a new command relationship to achieve it. Therefore, the transition from one activity to another when completing a given job task using the “task” “phase” variable in the Matlab structure. The “phase” variable is updated when the previous Action completes the desired precision. The task update phase starts when the UVMS approaches the desired M path point navigation target position. The “UpdateMissionPhase” of each loop performed the phase update condition check.

**Figure 9:**
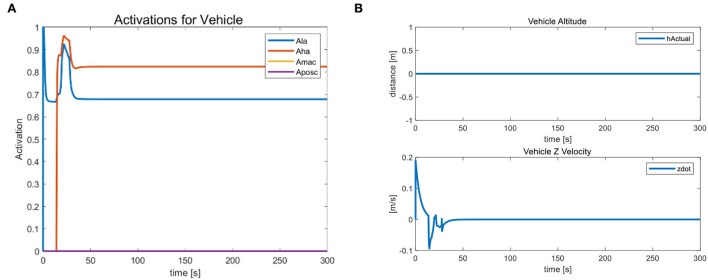
**(A)** The task activation status used for action transitions and **(B)** the behavior of the landing task.

As shown in Figure 9, by taking into account the buffer time, achieving a seamless transition (from one activity to another) is by using a bell curve (increasing or decreasing) activation. The transition triggers Actions A and B by realizing the vehicle's target position. We calculate the Cartesian error between the target frame and the vehicle frame, and the task phase changes when the error is below a given threshold (0.1 m in this case). When we want to disable the running task, use a decreasing bell function to perform a smooth transition. In this case, the minimum height and vehicle position tasks are disabled at the beginning of the second phase.

Similarly, when we want to activate the task, a smooth transition is performed using an increasing bell function, as we did for the landing task. We calculate the slope of such a function based on the task phase time, which helps us determine the time interval between vehicles switching from one activity to another (0.2 s in our case). At the same time, we obtain a smoother shape and avoid discontinuities in the motor drive. Due to the active hold state of the landing task “LA” and the horizontal attitude task “HA”, the vehicle's altitude and velocity in the Z-axis direction remain constant during this process and continue to be 0. Figure 9B indicates that the vehicle does not float with the external during the task transition. The task prioritization strategy has a strong constraint, proving its stability.

### Case 2: Add an optimal control target

After the task transition is complete, begin completing a joint limits avoidance task. Attempt to reach the specified operating position using the end-effector. Moreover, we observe that the vehicle does not move and that all joints are within their soft restraint limits. This task is a safety task, so it has a higher priority than other tasks that define movements. It can control the operation of the joints without exceeding their fixed thresholds. The action is the same as before; the only difference is that the joint restraint is always active, as this is a safety task. It is essential to ensure that the end-effector performs the final operation. This case adds the optimization goal of keeping the four joints of the manipulator's end-effector to complete the trajectory optimized for the target behavior. For the rest of the tasks, we kept the same hierarchy as in the previous case and added only the optimization task “MP”. This task has the lowest priority because we can only perform trajectory optimization of the end-effector after the UVMS completes all actions.

**Figure 10:**
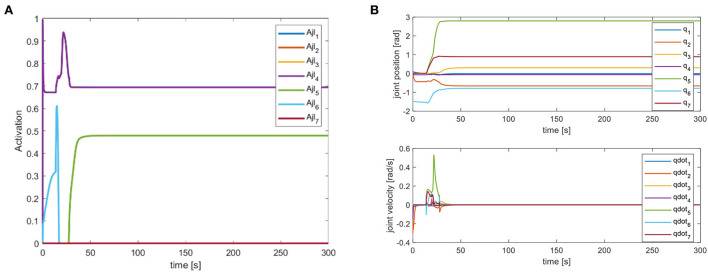
**(A)** Joint limitation task activation of action D. **(B)** Robot arm joint position and velocity variation curve.

**Figure 11:**
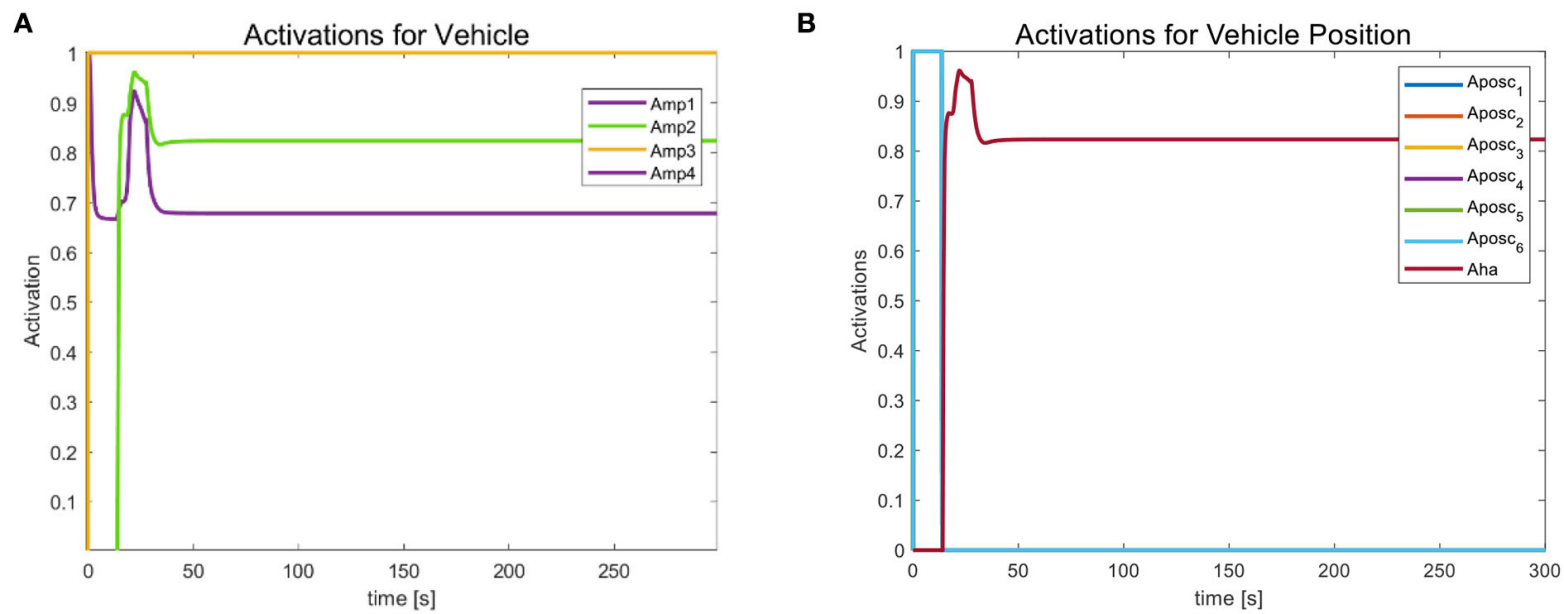
**(A)** Optimized task activation of the end-effector. **(B)** Task activation of the vehicle position.

**Figure 12:**
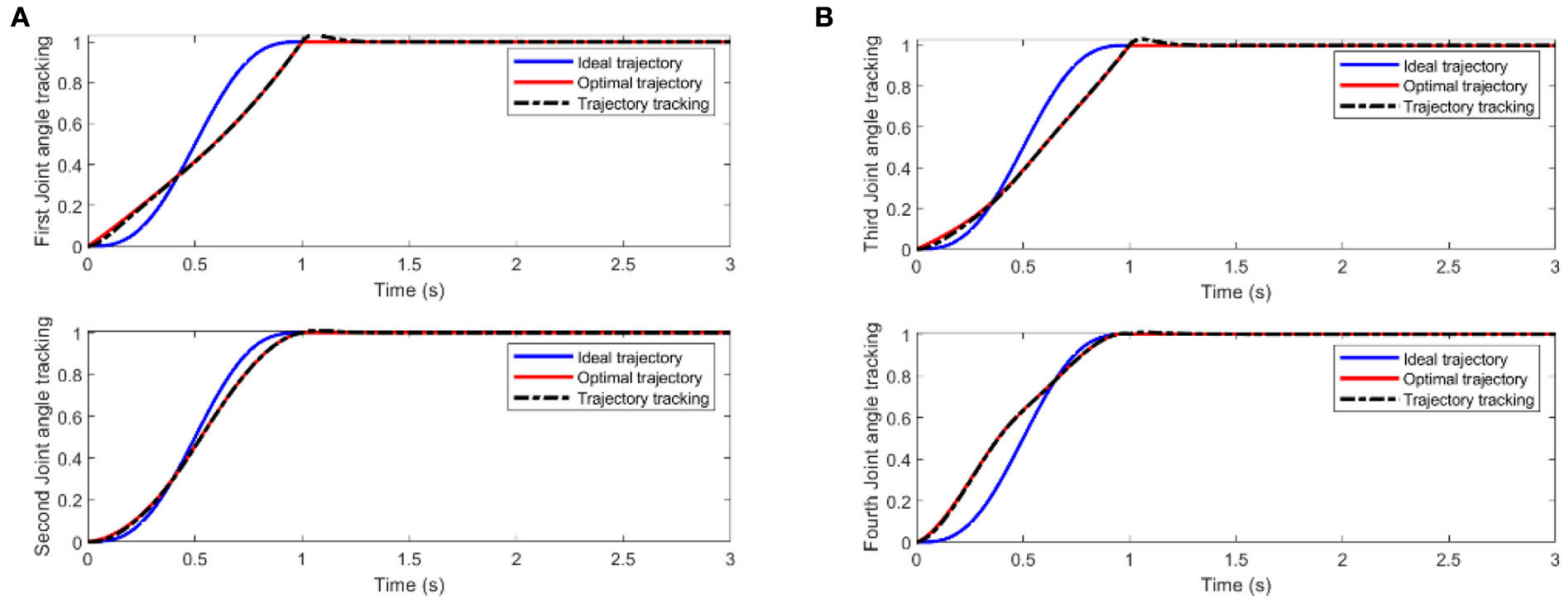
**(A)** Two-joint angular tracking of end-effector. **(B)** Two-joint angular tracking of end-effector.

We mainly activate the state of the four joints near the end of the end-effector, as shown in Figure 10A, and limit the motion of the remaining joints. The designation of the joint limit task is to test whether we can effectively control the activation state of each joint and motion-tracked it in real-time during the vehicle manipulation task. Figure 11 shows that while the end-effector optimization task is active, the horizontal attitude task “HA” is kept highly active to maintain the stability of the vehicle position. Figure 12 shows the optimal end-effector trajectory based on the trajectory optimization objective. The trajectory optimization is performed based on the satisfaction of the proposed constraints, and the desired optimal trajectory of the end-effector coincides with the trajectory tracking as much as possible. We ensure the accuracy and idealization of the task execution. In addition, Figure 12A shows the joint motion and Figure 12B is smoother compared to Figure 10B. The optimal solution here satisfies the primary collision-free motion task, meaning that the solution found here satisfies the primary collision-free task but is optimal compared to the suboptimal pose task. Therefore, the pure QP process cannot handle more than one task simultaneously, so we have placed the optimization task at the lowest priority.

## Conclusions

This paper studied the problem of multiple-task planning from the motion planning level for the underwater vehicle manipulator system. The task prioritization strategy to perform various tasks at once is considered in the mission planning to derive an optimal and feasible planning scheme; secondly, the optimization algorithm is adopted during the execution of the tasks, considering the system's limitations and the interference of the environment. We proposed soft constraints as an improved collision avoidance method to add more conditions to smooth the joint trajectory. The combination of the above two aspects can achieve the continuous planning of the phased execution of the task, ensure the stability of the end-effector work, and improve the reliability of UVMS autonomous underwater operations. We perform a series of simulations in a simulation environment established by kinematic and dynamic analysis of the underwater vehicle manipulator system. The simulation results verify the effectiveness and feasibility of this paper's task prioritization processing strategy. In this sense, we believe that the approach of using a simulation environment instead of a natural underwater application environment proves to be cost-saving in planning and effective in improvement. And in this sense, we believe that our work has achieved some progress in extending the scope of applying task prioritization planning methods based on the motion planning level in control studies of UVMS. In addition, our proposed method provides an optional way of thinking for controlling underwater robots and other types of robots. In the future, we plan to extend the research for different kinds of robots for real-time planning.

Indeed, the current research has its limitations, along with some results. In the algorithm proposed in this research, we run the simulations carried out under ideal conditions, so it needs to complete realistic experiments to verify the correctness and feasibility of the proposed method. In addition, more influencing factors should be considered, such as the reliability of the sensor, actuator, and controller execution methods. Moreover, adding and improving the controller's performance and stability to accomplish the smooth execution of the task is an essential topic for further research. Ultimately, UVMS-related research has broad application background and important theoretical and engineering significance. Our proposed method will be applied to UVMS for autonomous motion planning in unknown sea environments to enhance its subsea operation capability and meet the application requirements of keeping up with the times.

## Data availability statement

The original contributions presented in the study are included in the article/supplementary material, further inquiries can be directed to the corresponding author.

## Author contributions

Conceptualization: Y-eG and XZ. Methodology, writing—original draft preparation, writing—review and editing, software, formal analysis, and data curation: Y-eG. Validation: WB, JW, and QY. Supervision, project administration, and funding acquisition: WB, SY, and YS. All authors have read and agreed to the published version of the manuscript.

## Funding

This research was funded by the Basic Research in Natural Science and Enterprise Joint Fund of Shaanxi (No. 2021JLM-58).

## Conflict of interest

All authors were employed by company Hanjiang-Weihe River Valley Water Diversion Project Construction Co., LTD.

## Publisher's note

All claims expressed in this article are solely those of the authors and do not necessarily represent those of their affiliated organizations, or those of the publisher, the editors and the reviewers. Any product that may be evaluated in this article, or claim that may be made by its manufacturer, is not guaranteed or endorsed by the publisher.
